# IGFBP-6 regulates breast cancer cell cycle progression by promoting exit out of G1

**DOI:** 10.1016/j.jbc.2025.111069

**Published:** 2025-12-17

**Authors:** Francisco J. Lariz, Shayla Hernandez, Diana C. Bautista-Tovar, Kevin D. Houston

**Affiliations:** Department of Chemistry and Biochemistry, New Mexico State University, Las Cruces, New Mexico, USA

**Keywords:** IGFBP-6, G2/M phase, cell cycle, interferon, cyclin B1, breast cancer

## Abstract

While the contribution of the IGF-signaling axis in breast cancer is well-documented, the role of IGFBP-6 in breast carcinogenesis has not been extensively studied. In general, insulin-like growth factor binding protein-6 (IGFBP-6) sequesters insulin-like growth factor 2 (IGF-2) to attenuate activation of its cognate receptor IGF-1R. To reveal previously unknown mechanisms of breast cancer modulation by IGFBP-6 in breast cancer, proteomic analysis was performed in T47D cells after IGFBP-6 knockdown. Comparing protein expression and phosphosites after knockdown by unique siRNA sequences with a negative control and subsequent pathway analysis, a decrease in IGFBP-6 expression resulted in activation of interferon signaling pathways and a decrease in pathways involved in the G2/M cell cycle transition. A subset of the proteins identified in each cell regulatory pathway was validated by immunoblotting for specific proteins after IGFBP-6 knockdown. Cell cycle analysis showed that IGFBP-6 knockdown in Hormone Receptor Positive T47D breast cancer cells resulted in an increased number of cells in the G1 phase and a decrease in cells in G2, indicating a role for IGFBP-6 in cell cycle regulation. Knockdown of IGFBP-6 in a triple-negative breast cancer cell line, MDA-MB-231, also resulted in a decrease in Cyclin B1 accumulation, demonstrating that our observations are not cell line specific. Taken together, our results demonstrate that IGFBP-6 regulates cell cycle progression in breast cancer cells and interferon signaling in hormone-positive cells.

The regulation of breast cancer by the IGF-signaling axis is well documented. Insulin-like growth factor binding protein-6 (IGFBP-6), a component of the IGF signaling axis, is associated with improved outcomes in breast cancer patients (1, 2, 3). However, IGFBP-6 has not been extensively studied in breast cancer, and the molecular mechanisms of breast cancer cell regulation by IGFBP-6 remain mostly unknown. IGFBP-6 is one of six IGFBPs that bind to insulin-like growth factors 1 or 2 (IGF-1 or 2), increasing IGF half-life and attenuating binding to the IGF-1 receptor (IGF-1R) (4, 5, 6). Activation of IGF-1R promotes survival and proliferation in breast cancer through activation of the MAPK and PI3K pathways (reviewed in (6)). IGFBP-6 uniquely binds IGF-2 with a 20-50-fold affinity over IGF-1 and has been considered an exclusive inhibitor of IGF-2 (7). IGFBP-6 has functions independent of IGF-2, including regulating migration (8), modulating inflammation (9, 10) DNA repair (11) and delaying senescence (12). IGFBP-6 appears to have a tumor suppressive role, as many breast cancers have lower expression of IGFBP-6 compared to normal tissue (1). IGFBP-6 has been associated with lactate metabolism (13) and cell migration in breast cancer cells (14). Prior work has demonstrated that IGFBP-6 is induced by progesterone in breast cancer cells and modulates progesterone signaling (3). However, the molecular mechanisms of breast cancer cells regulation by IGFBP-6 have not been determined.

Proteomic analysis of breast cancer cells after siRNA-mediated knockdown of IGFBP-6 identified a potential role of IGFBP-6 in the regulation of interferon signaling and cell cycle progression. These data revealed upregulated phosphosites associated with interferon signaling and downregulated phosphosites associated with cell cycle progression. Additionally, upregulated proteins were identified as interferon-stimulated genes (ISGs) and the downregulation of proteins associated with the G2/M phases of the cell cycle were observed in breast cancer cells with reduced IGFBP-6. Furthermore, Gene Set Enrichment Analysis identified that the most upregulated pathways were associated with interferon signaling and the most downregulated pathways were associated with regulation of the G2/M phase of the cell cycle.

Based on these results, the role of interferon signaling and cell cycle dysregulation in breast cancer was further studied. Interferon signaling is part of innate immunity in response to viral infection or in response to genotoxic stress or chromosomal instability (15). Type 1 interferon signaling is associated with interferon alpha or beta (IFN α or β), which activates the JAK-STAT pathway (15, 16). In response to damage-associated molecular pattern molecules (DAMPs), type 1 interferons can initiate pathways which destroy cancer cells (15, 16, 17). However, low, chronic levels of interferon can result in an Interferon-Related DNA Damage Signature (IRDS) which can confer resistance to chemotherapy and radiation (16, 18, 19). An IRDS response involves the expression of a subset of interferon stimulated genes (ISGs) which can promote the survival of cancer cells which can generally evade apoptosis (16, 18, 19). Interferon signaling can also cause arrests in the cell cycle or entry into senescence as reviewed by Qin, 2023 (20). The cell cycle is regulated by a series of checkpoints which can become compromised in cancer (reviewed in (21)). Disruption of the cell cycle or blockade of cell cycle phases can activate interferon signaling (22, 23, 24).

The findings from the proteomic analysis were validated and the role of IGFBP-6 in cell cycle regulation and resistance to radiation were determined. Knockdown of IGFBP-6 resulted in an increase in cells in the G1 phase and a decrease in cells in the G2 phase. Cell proliferation increased in IGFBP-6 low cells despite changes in cell cycle phase distributions. To test for an IRDS response, T47D cells were irradiated with a dose of 4 Gy which resulted in increased recovery. In Triple Negative MDA-MB-231 cells, knockdown of IGFBP-6 also resulted in decreases in Cyclin B1 levels but no increases in IFNb expression or resistance to irradiation. Taken together, IGFBP-6 facilitates exit out of G1 and confers resistance to irradiation from activation of interferon signaling.

## Results

### Knockdown of IGFBP-6 induced interferon-stimulated genes (ISGs) and decreases proteins associated with G2/M phase

**Figure 1:**
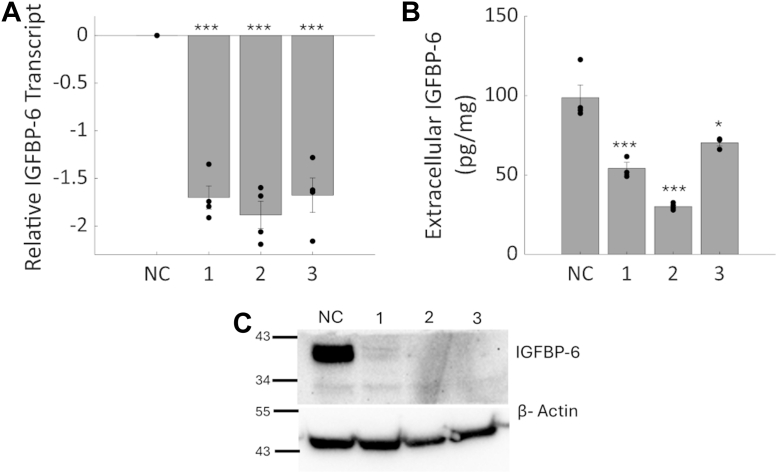
**Knockdown of IGFBP-6 in T47D cells.***A*, IGFBP-6 Transcript levels measured by qPCR. N = 4. *B*, secreted IGFBP-6 levels measured by ELISA. Units are pg of IGFBP-6/mg of extracellular protein. N = 3. *C*, representative Intracellular IGFBP-6 Western Blot N = 5. *Asterisks* indicated significance level. ∗ means *p* < 0.05, ∗∗ means *p* < 0.01, and ∗∗∗ means *p* < 0.001.

**Figure 2:**
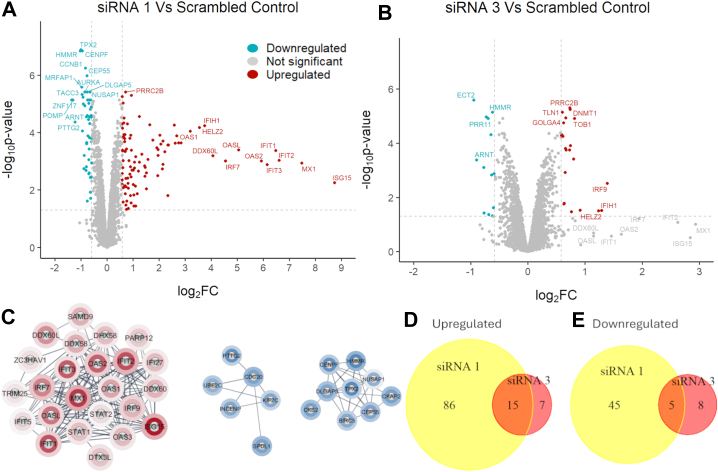
**Proteomics results.***A*, Volcano plot displaying differentially expressed proteins in cells treated with siRNA one relative to the scrambled negative control. *B*, Volcano plot displaying differentially expressed proteins in cells treated with siRNA three relative to the scrambled negative control. *C*, Markov cluster algorithm results for differentially expressed proteins. The inner ring corresponds to results from siRNA one and the outer ring corresponds to the result from siRNA 3. *Red* is for upregulated proteins and *blue* is for downregulated proteins. *D*, Venn diagram displaying agreement in upregulated proteins between each siRNA treatment relative to the negative control. *E*, Venn diagram displaying agreement in downregulated proteins between each siRNA treatment relative to the negative control.

A prior study identified IGFBP-6 as a progesterone responsive gene and demonstrated that knockdown of IGFBP-6 altered response to progesterone in breast cancer cells (3). To further study how IGFBP-6 regulates breast cancer cells, IGFBP-6 levels were knocked down using three unique siRNA sequences. This approach resulted in a significant decrease in IGFBP-6 transcript, and both extracellular and intracellular IGFBP-6 protein. (Fig. 1). SiRNAs one and three were selected for further analysis by proteomics since these sequences were used in the prior study. Proteomic analysis after IGFBP-6 knockdown identified (Fig. 2) 6309 unique proteins, with 86 proteins significantly upregulated, and 50 were downregulated after treatment with siRNA 1. In siRNA 3, 22 proteins were significantly upregulated and 13 downregulated. When analyzed together, 15 proteins were upregulated in both siRNA treatments and five were downregulated (Fig. 2*D* and e, Table 1). The full list of differentially expressed proteins are provided in Tables S1 and S2. Similar changes in differential protein expression were observed in both siRNA sequences, but siRNA three had smaller changes in protein expression relative to the scrambled negative control. The results related to quality control are displayed in Figs. S1 and S2. All colored points on the volcano plots refer to proteins which are above the 1.5 fold threshold and that have an adjusted *p* value less than 0.05.

**Table 1:** Differentially expressed proteins agreed by both siRNA treatments

UniProt ID	Protein	Description	Log_2_ FC siRNA 1	adj.P.Val siRNA 1	Log_2_ FC siRNA 3	adj.P.Val siRNA 3
Upregulated						
Q9BYX4	IFIH1	Interferon-induced helicase C domain-containing protein 1	3.754	5.727E-05	1.291	3.095E-02
Q9BYK8	HELZ2	3–5 exoribonuclease HELZ2	3.551	6.776E-05	1.240	3.180E-02
Q00978	IRF9	Interferon regulatory factor 9	2.592	5.524E-05	1.392	3.062E-03
Q8TDB6	DTX3L	E3 ubiquitin-protein ligase DTX3L	2.032	1.253E-04	0.762	3.452E-02
P10321	HLA-C	HLA class I histocompatibility antigen, C alpha chain	1.961	3.950E-04	0.917	3.029E-02
P19525	EIF2AK2	Interferon-induced, double-stranded RNA-activated protein kinase	1.645	5.343E-05	0.633	1.747E-02
P50616	TOB1	Protein Tob1	0.944	5.062E-06	0.817	1.223E-05
Q5W0Z9	ZDHHC20	Palmitoyltransferase ZDHHC20	0.879	1.583E-04	0.798	3.841E-04
O15126	SCAMP1	Secretory carrier-associated membrane protein 1	0.785	5.048E-05	0.671	1.817E-04
Q9Y6B6	SAR1B	Small COPII coat GTPase SAR1B	0.758	3.791E-04	0.651	1.224E-03
Q5JSZ5	PRRC2B	Protein PRRC2B	0.724	3.852E-06	0.737	4.999E-06
Q13740	ALCAM	CD166 antigen	0.680	1.639E-04	0.747	1.226E-04
Q04917	YWHAH	14-3-3 protein eta	0.655	3.116E-05	0.613	5.600E-05
Q9NV35	NUDT15	Nucleotide triphosphate diphosphatase NUDT15	0.637	1.392E-04	0.657	1.575E-04
P26358	DNMT1	DNA (cytosine-5)-methyltransferase 1	0.624	9.387E-06	0.742	5.699E-06


UniProt ID	Protein	description	logFC_1	adj.P.Val_1	logFC_3	adj.P.Val_3
Downregulated						
Q9Y244	POMP	Proteasome maturation protein	−1.302	7.305E-06	−0.592	1.362E-03
P27540	ARNT	Aryl hydrocarbon receptor nuclear translocator	−1.224	4.322E-05	−0.896	4.153E-04
O75330	HMMR	Hyaluronan mediated motility receptor	−1.021	1.420E-07	−0.620	7.205E-06
P78524	DENND2B	DENN domain-containing protein 2B	−0.748	2.432E-02	−0.685	4.295E-02
Q96HE9	PRR11	Proline-rich protein 11	−0.607	2.560E-05	−0.730	1.069E-05

Proteomic analysis identified induction of interferon-stimulated genes (ISGs) relative to the scrambled negative control. These include Interferon regulatory factor 9 (IRF9), Interferon-Inducible Helicase Containing Protein 1 (IFIH1), HELZ2, and Interferon-Induced, Double-Stranded RNA-Activated Protein Kinase (EIF2AK2). IRF9 is a component of the ISGF3 transcriptional complex which promotes the expression of ISGs (15, reviewed in (25)). IFIH1 (also known as MDA-5) is an RNA sensor that detects long RNA fragments to induce IFNb (reviewed in (26)). HELZ2 is a 3′-5′ exonuclease that has been identified as an ISG (27). EIF2AK2 (also known as PKR) is an interferon-inducible kinase that can be activated in response to double-stranded RNA to block translation of viral components (28). In cells treated with siRNA 1, the RNA sensors EIF2AK2, IFIH, and RIG-I are upregulated, suggesting that they could contribute to the increased interferon-response relative to siRNA 3. It was determined that the intracellular IGFBP-6 intracellular protein was knocked down effectively, but extracellular knockdown of IGFBP-6 varied based on the siRNA used. siRNA3 had the lowest decrease in extracellular IGFBP-6, which could explain why the response was weakened compared to siRNA 1.

Downregulated proteins include Proteosome Maturation Protein (POMP), Aryl Hydrocarbon Receptor Nuclear Translocator (ARNT or HIF1b), and Hyaluronan Mediated Motility Receptor (HMMR or RHAMM). POMP is associated with the assembly of the proteasome (29). ARNT (also known as HIF1b) is associated in response to hypoxia and associates with HIF1a as well as the Aryl hydrocarbon receptor and Estrogen receptor alpha (30). HMMR is a hyaluronic acid receptor that is associated with both cell migration and mitotic spindle formation (31).

**Figure 3:**
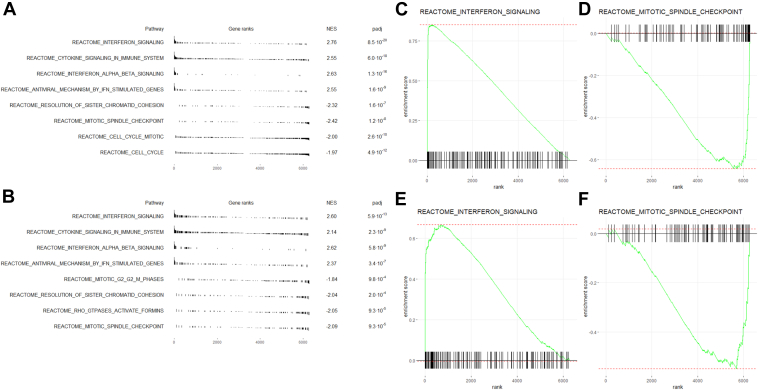
**Gene set enrichment analysis results.***A*, enrichment table for siRNA one relative to the scrambled control. *B*, enrichment table for siRNA one relative to the scrambled control. *C*, enrichment plot for the Interferon Signaling Pathway for siRNA one relative to the scrambled control. *D*, enrichment plot for the Mitotic Spindle Checkpoint Pathway for siRNA one relative to the scrambled control. *E*, enrichment plot for the Interferon Signaling Pathway for siRNA three relative to the scrambled control. *F*, enrichment plot for the Mitotic Spindle Checkpoint Pathway for siRNA three relative to the scrambled control.

Protein interaction networks were generated using Cytoscape (Fig. 2*C*), and Gene Set Enrichment Analysis was performed to identify signaling pathways that are potentially regulated by IGFBP-6 (Fig. 3). The most upregulated pathways are associated with interferon signaling and antiviral responses (Fig. 3, *A* and *D*), while the most downregulated pathways were associated with the G2/M phases of the cell cycle and the mitotic spindle checkpoint. These results suggest that the IGFBP-6 regulates interferon signaling and the G2/M phase of the cell cycle.

### Knockdown of IGFBP-6 alters levels of phosphopeptides associated with interferon signaling and the cell cycle

**Figure 4:**
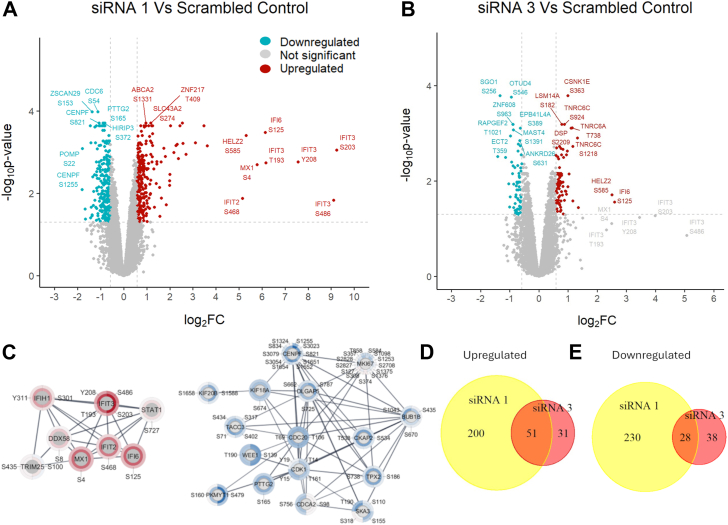
**Phosphoproteomics results.***A*, Volcano plot displaying differentially expressed phosphopeptides in cells treated with siRNA one relative to the scrambled negative control. *B*, Volcano plot displaying differentially expressed phosphopeptides in cells treated with siRNA three relative to the scrambled negative control. *C*, Markov cluster algorithm results for differentially expressed phoshopeptides. The inner ring corresponds to results from siRNA one and the outer ring corresponds to the results from siRNA 3. *Red* is for upregulated proteins, and *blue* is for downregulated phosphopeptides. *D*, Venn diagram displaying agreement in upregulated phosphopeptides between each siRNA treatment relative to the negative control. *E*, Venn diagram displaying agreement in downregulated phosphopeptides between each siRNA treatment relative to the negative control.

Phosphoproteomics was also performed because IGFBP-6 is known to attenuate IGF-2 signaling through IGF-1R (1, 2, 3) and downstream phospho-dependent regulation of signal transduction pathways such as MAPK- and AKT-dependent cell signaling pathways. In total, 7223 phosphosites were identified. Relative to the scrambled negative control, treatment with siRNA one resulted in upregulation of 251 phosphosites and downregulation of 258 phosphosites (Fig. 4*A* and Table S3). With siRNA 3, 82 phosphosites were upregulated and 66 were downregulated (Fig. 4*B* and Table S4). 51 significantly upregulated phosphosites and 28 significantly downregulated phosphosites were present after treatment with both siRNAs (Fig. 4, *D* and *E*, Tables 2, and 3). Many of the differentially expressed phosphosites identified in siRNA one were similarly identified in siRNA three but did not exceed the threshold value of 1.5. Data related to quality control are displayed in Figs. S3 and S4. These phosphosites were generally not indicative of increased relative phosphorylation but instead the result of increased expression of each of these peptides. Phosphosites identified in siRNA one were similarly identified in siRNA three but did not exceed the threshold value.

**Table 2:** Most upregulated phosphopeptides

UniProt ID	Protein	Description	Position	logFC_2_ siRNA 1	adjPVal siRNA 1	logFC_2_ siRNA 3	adjPVal siRNA 3
P09912	IFI6	Interferon alpha-inducible protein 6	S125	6.151	3.337E-04	2.600	2.795E-02
Q9BYK8	HELZ2	3′-5′ exoribonuclease HELZ2	S585	5.311	3.985E-04	2.511	1.970E-02
Q9BYK8	HELZ2	3′-5′ exoribonuclease HELZ2	S2268	2.707	1.131E-03	1.359	3.617E-02
Q9HCJ0	TNRC6C	Trinucleotide repeat-containing gene 6C protein	S1218	1.171	1.131E-03	1.323	1.254E-03
Q07002	CDK18	Cyclin-dependent kinase 18	S98	2.174	1.018E-03	1.167	2.618E-02
Q13439	GOLGA4	Golgin subfamily A member 4	S89	0.877	3.476E-03	1.164	1.901E-03
Q8NDV7	TNRC6A	Trinucleotide repeat-containing gene 6A protein	T738	1.259	2.302E-04	1.147	7.751E-04
P15924	DSP	Desmoplakin	S2209	1.215	2.311E-04	1.101	7.793E-04
Q6DN90	IQSEC1	IQ motif and SEC7 domain-containing protein 1	S417	0.734	2.148E-02	1.038	7.135E-03
Q9Y519	TMEM184B	Transmembrane protein 184B	S388	1.301	2.552E-03	1.000	1.325E-02
Q8NDV7	TNRC6A	Trinucleotide repeat-containing gene 6A protein	S991	1.161	5.348E-04	0.972	2.361E-03
Q8TEY5	CREB3L4	Cyclic AMP-responsive element-binding protein 3-like protein 4	S109	0.869	2.043E-02	0.947	1.921E-02
O43164	PJA2	E3 ubiquitin-protein ligase Praja-2	S80	0.883	1.312E-02	0.902	1.631E-02
Q9HCJ0	TNRC6C	Trinucleotide repeat-containing gene 6C protein	S924	0.903	2.480E-04	0.879	6.492E-04
P98175	RBM10	RNA-binding protein 10	S687	1.177	4.803E-03	0.877	2.556E-02
O75362	ZNF217	Zinc finger protein 217	S848	1.121	4.154E-04	0.843	3.231E-03
Q5JSZ5	PRRC2B	Protein PRRC2B	S226	1.018	1.752E-02	0.833	4.957E-02
Q8NE35	CPEB3	Cytoplasmic polyadenylation element-binding protein 3	S192	1.063	1.877E-03	0.825	1.055E-02
O75362	ZNF217	Zinc finger protein 217	S407	1.237	1.310E-03	0.824	1.304E-02

**Table 3:** Most downregulated phosphopeptides

UniProt ID	Protein	Description	Position	logFC siRNA 1	adjPVal siRNA 1	logFC siRNA 3	adjPVal siRNA 3
Q71RC2	PSMG1	Proteasome assembly chaperone 1	T18	−1.451	2.302E-04	−0.889	4.852E-03
Q5FBB7	PALM3	Paralemmin-3	S155	−1.428	5.960E-04	−0.628	3.632E-02
P30291	SKA3	Spindle and kinetochore-associated protein 3	T190	−1.185	1.743E-03	−0.730	2.364E-02
Q8IWZ3	LARP4	La-related protein 4	S647	−1.181	3.656E-03	−1.414	3.099E-03
Q9ULD9	H2AX	Histone H2AX	T121	−1.175	7.450E-03	−0.869	3.639E-02
Q8IWZ3	TOP2A	DNA topoisomerase 2-alpha	S1332	−1.064	1.743E-03	−0.638	2.621E-02
O95456	BUB1	Mitotic checkpoint serine/threonine-protein kinase BUB1	S655	−1.063	6.938E-04	−0.827	5.265E-03
Q9ULD9	WEE1	Wee1-like protein kinase	S139	−1.026	2.700E-03	−1.144	3.231E-03
P16104	ANKHD1	Ankyrin repeat and KH domain-containing protein 1	S1679	−1.026	1.298E-02	−0.983	2.150E-02
O43683	ANKHD1	Ankyrin repeat and KH domain-containing protein 1	S1670	−1.022	2.308E-03	−0.908	7.065E-03
P48634	CDCA7L	Cell division cycle-associated 7-like protein	S117	−1.003	6.782E-04	−0.666	8.433E-03
Q15154	ZNF608	Zinc finger protein 608	S963	−0.995	2.302E-04	−0.886	6.492E-04
P51114	TOP2A	DNA topoisomerase 2-alpha	S1354	−0.989	3.722E-03	−0.706	2.504E-02
Q5VTR2	ZNF608	Zinc finger protein 608	S627	−0.982	1.591E-03	−0.932	4.013E-03
Q15154	PRRC2A	Protein PRRC2A	S1219	−0.949	1.880E-02	−0.801	4.720E-02
Q8IX90	CDC20	Cell division cycle protein 20 homolog	T69	−0.846	4.567E-03	−0.702	1.547E-02
Q96DX7	FXR1	RNA-binding protein FXR1	S406	−0.839	1.056E-02	−0.784	1.970E-02
P11388	POC5	Centrosomal protein POC5	S109	−0.800	1.140E-02	−0.662	3.387E-02
P06400	RNF20	E3 ubiquitin-protein ligase BRE1A	S525	−0.780	3.198E-02	−0.764	4.562E-02
Q12834	CDK1	Cyclin-dependent kinase 1	Y15	−0.776	9.337E-04	−0.621	5.358E-03
P11137	PCM1	Pericentriolar material 1 protein	S69	−0.756	2.574E-02	−0.732	3.893E-02
Q96GN5	PCM1	Pericentriolar material 1 protein	S1958	−0.737	4.032E-02	−0.786	4.126E-02

The most upregulated phosphopeptides were IFI6 S125, HELZ2 at S585 and S2268, CDK18 S98, and TMEM184B at S388. IFI6 is an ISG which is a negative regulator of interferon signaling (32). HELZ2 had increased phosphorylation at two sites, S585 and S2268. Cyclin-dependent kinase 18 (CDK18) was another upregulated phosphopepetide at S98. CDK18 is associated with promoting DNA-damage repair (33). TMEM184B has been associated with promoting aggressiveness in hypopharyngeal squamous cell carcinoma (34).

**Figure 5:**
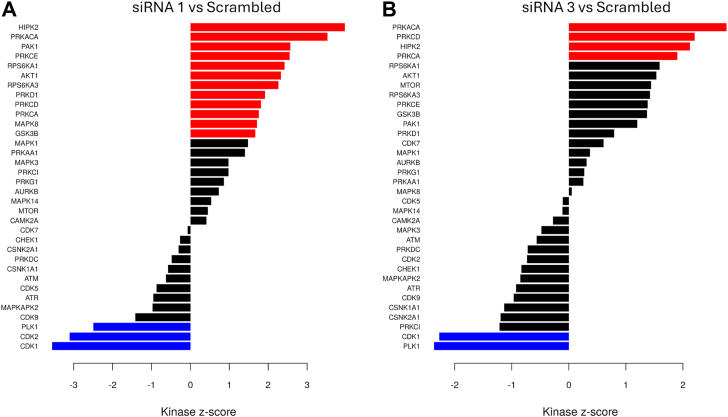
**Kinase-substrate enrichment analysis results.***A*, predicted Kinase activity for siRNA 1. *B*, predicted kinase activity for siRNA 3. *Red* indicates kinases whose activity is predicted to be upregulated. *Blue* indicates kinases whose activity is predicted to be downregulated.

The most downregulated phosphopeptides included PSMG1 at T18, PALM3 at S155, SKA3 at T190, LARP4 at S647, and H2AX T121. PSMG1 is associated with proteasome assembly and when associated with NUP37 produces worse recurrence-free survival and distant metastasis survival in breast cancer (35). Many of the downregulated phosphosites are substrates of mitotic regulators CDK1, Polo-like Kinases, Aurora Kinases (36). Cyclin-dependent kinase 1 (CDK1) had decreased phosphorylation at a key regulatory site Y15, which is a target of WEE1 (37), and at T14, which is a target of PKMYT1 (38). These regulatory sites inhibit the activity of the cyclin B1-CDK1 complex and slow entry into mitosis (39). Wee1 and CDK1 enable are regulated by a feedback loop where active Cyclin B1-CDK1 can phosphorylate and degrade Wee1 (40). Wee1 phosphorylation was decreased at S139 and T190. Wee1 phosphorylation at S139 is associated with regulating the stability of Wee1 (40). Protein networks and phosphosites were visualized using CytoScape and suggested a role for IGFBP-6 in the regulation of interferon signaling and cell cycle progression (Fig. 4*E*). Each node has two donut plots with the inner ring representing siRNA one and the outer ring to siRNA 3. To analyze altered pathways, Kinase-Substrate Enrichment analysis was done using KSEAapp (Fig. 5) HIPK2, PRKACA, and PRKCD had the greatest increases in kinase activity, and CDK1 and PLK1 had the most decreased activity. The elevated HIPK2 activity is likely the result of elevated interferon signaling as HIPK2 is necessary in carrying out type 1 interferon responses (41). PRKACA is a catalytic subunit of protein kinase A (42). PRKCD or protein kinase c-δ is activated by type 1 interferons (43). These results indicate that knockdown of IGFBP-6 results in increased levels of interferon-associated phosphorylation and decreased levels of phosphoproteins associated with G2/M. Taken together, both the proteomics and phosphoproteomics analyses support activation of interferon signaling and decreases in G2/M entry in T47D cells.

### Interferon beta is induced by IGFBP-6 knockdown in T47D cells

**Figure 6:**
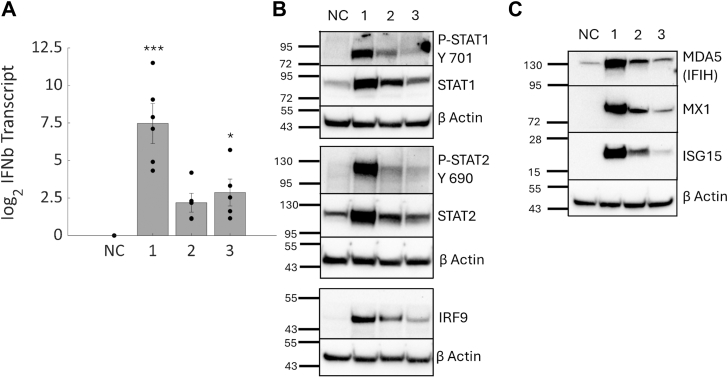
**Verification of interferon signaling activation.***A*, interferon Beta (IFNb) transcriptional levels relative to the scrambled negative control. N = 5. *B*, phosphorylation and Expression of components of the ISGF3 complex. N = 6 to 8 for each blot. *C*, representative Western blot for ISGs identified from proteomics. N = 8 for each. ∗ means *p* < 0.05, ∗∗ means *p* < 0.01, and ∗∗∗ means *p* < 0.001.

To validate the results obtained in the proteomic analysis, IGFBP-6 was knocked down with siRNA and interferon beta (IFNb) transcript was measured. IFNb mediates Type 1 interferon response *via* activation of the ISGF3 complex composed of STAT1, STAT2, and IRF9 (15). Relative to the scrambled negative control, knockdown of IGFBP-6 resulted in an induction of IFNb (Fig. 6*A*), with the greatest induction observed with siRNA 1. Additionally, the expression of components of the ISGF3 complex exhibits increased expression and phosphorylation (Fig. 6*B*). STAT1 has significantly increased phosphorylation at Y701 after knockdown with siRNA one and 2. siRNA three also has elevated but nonsignificant phosphorylation of STAT1. STAT1 expression is significantly higher after treatment with siRNA 1. STAT2 has significantly increased phosphorylation at Y690 with siRNA one and elevated but nonsignificant increases in expression. IRF9 levels were significantly increased in all IGFBP-6 knockdowns. A few ISGs identified in the proteomics were also validated and had increased expression of IFIH, MX1, and ISG15 (Fig. 6*C*). These results validate that increased expression of ISGs is due to increased activation of Type 1 Interferon signaling.

### Knockdown of IGFBP-6 changes cell cycle distributions and confers radiation resistance

**Figure 7:**
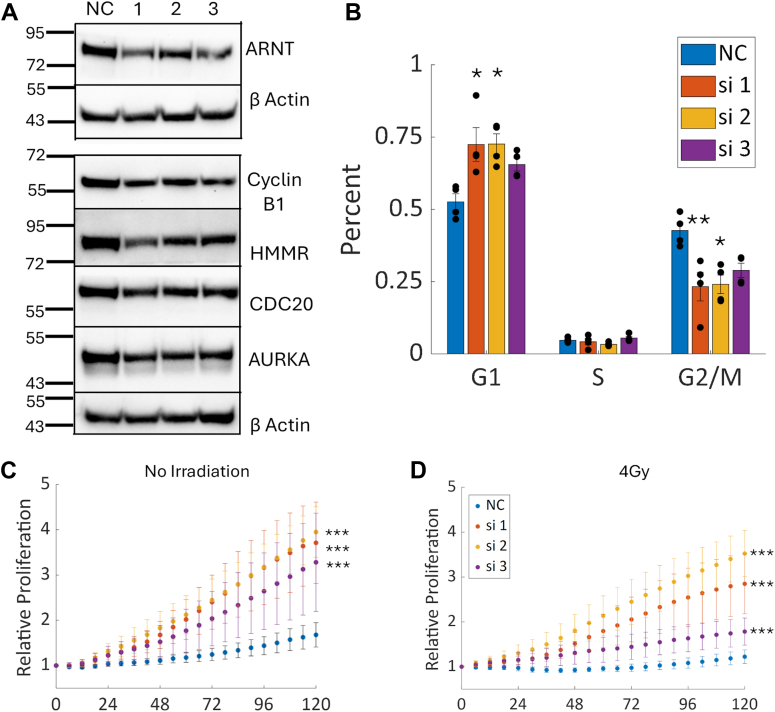
**Verification of decreased g2/m protein expression and cell cycle changes.***A*, representative western blots for downregulated proteins after IGFBP-6 knockdown. *B*, FUCCI reporter results after IGFBP-6 knockdown. N = 4. *C*, cell Proliferation after IGFBP-6 Knockdown. N = 4. *D*, cell proliferation after IGFBP-6 knockdown and 4Gy x-ray irradiation. N = 4. ∗ means *p* < 0.05, ∗∗ means *p* < 0.01, and ∗∗∗ means *p* < 0.001.

Downregulated peptides in the proteomic analysis belonged to proteins associated with the G2/M phases of the cell cycle, including ARNT, Cyclin B1, HMMR, CDC20, and AURKA (Fig. 7*A*). To validate that IGFBP-6 regulates cell cycle progression, T47D cells were transfected with a FUCCI Reporter system and changes in cell cycle phase distributions were measured. Knockdown of IGFBP-6 increased the percentage of cells in G1 and decreased the percentage of cells in G2/M (Fig. 7*B*). At most, 5% of cells were in S phase when measured, and there were no significant differences in the percentage of cells in S phase. Representative images of the T47D Fucci cells are shown in Fig. S5.

Cancer cells can utilize type 1 Interferons to promote survival and resistance to DNA damage in a state known as an Interferon-Related DNA Damage Signature (IRDS) involving the expression of low, chronic levels of IFNb and expression of a subset of ISGs (15, 16, 17, 18, 19). Since knockdown of IGFBP-6 results in increased expression of components of the ISGF3 complex and increased expression of interferon stimulated genes, it was hypothesized that these cells would resist irradiation. Additionally, cells in the G1 or S phase of the cell cycle are more likely to survive irradiation (44). Following IGFBP-6 knockdown, T47D cells were irradiated with a 4Gy dose of x-rays as previously reported (45). Without irradiation, knocked down cells exhibited increased proliferation (Fig. 7*C*), consistent with published data in breast cancer cells (14) and demonstrative of an antiproliferative effect of IGFBP-6 in breast cancer cells. After irradiation, the cells treated with the scrambled siRNA negative control did not recover (Fig. 7*D*), while knockdown of IGFBP-6 exhibited the greatest recovery after irradiation. Representative images are displayed in Figs. S6 and S7. These results suggest that IGFBP-6 regulates cell cycle progression.

siRNA can induce activation of Interferon signaling by binding toll-like receptors or binding to RNA sensors (46). To rule out the possibility that siRNA exposure is inducing interferon signaling and altering cell cycle progression, cells were treated with 50 U/ml IFNb and cell cycle progression and accumulation of cell cycle regulating proteins were measured (Fig. S8). The results show that the activation of interferon signaling did not alter the levels of cell cycle-regulating proteins, and the distributions of the cell cycle phases were not significantly changed. These results suggest that IGFBP-6 knockdown results in changes to the regulation of the cell cycle, and it is not a result of the interferon response. The expression of the G2/M proteins did not correlate with IGFBP-6 expression (Figs. S9 and S10) in the cell lines tested, suggesting that these proteins may undergo IGFBP-6-dependent posttranscriptional regulation.

### Knockdown of IGFBP-6 in MDA-MB-231 cells results in a reduction of cyclin B1

Transcriptional IGFBP-6 levels were measured in other breast cancer cell lines and in HEK293T cells to determine relative IGFBP-6 expression (Fig. S9). The cell lines with the lowest transcriptional expression, when compared to T47D cells, were the MCF-7 and HEK293T cells, and the highest were the MDA-MB-231 cells. T47D-Y cells, a subclone of T47D cells that have lost progesterone receptor (47), had higher levels of intracellular IGFBP-6 despite having lower transcriptional expression of IGFBP-6 compared to T47D cells. MDA-MB-468 cells and HEK293T cells had the greatest intracellular expression of IGFBP-6. The MDA-MB-231 cells were the highest expressing cell line both transcriptionally and extracellularly. Levels of extracellular IGFBP-6 followed the same pattern as the transcriptional levels. Intracellular levels of IGFBP-6 did not follow the same pattern as extracellular IGFBP-6.

**Figure 8:**
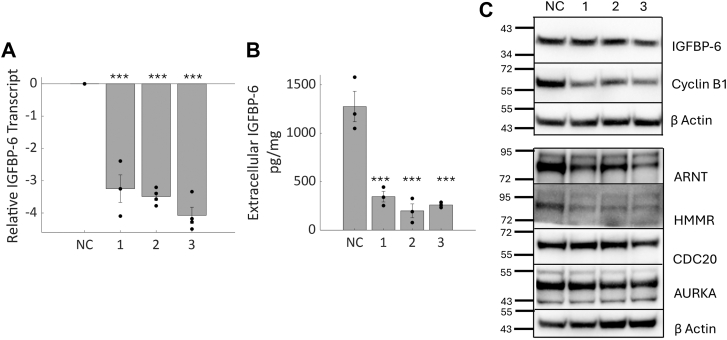
**IGFBP-6 knockdown in MDA-MB-231 cells.***A*, IGFBP-6 Transcript levels measured by qPCR. N = 4. *B*, secreted IGFBP-6 levels measured by ELISA. Units are pg of IGFBP-6/mg of extracellular protein. N = 3. *C*, representative Western blots of intracellular IGFBP-6 and proteins that were identified to decrease in the proteomics data sets. N = 5. ∗∗∗ means *p* < 0.001.

To test whether the changes observed in T47D cells were applicable to other breast cancer cell lines, knockdown of IGFBP-6 was done in MDA-MB-231 cells. A 90% knockdown was achieved in the MDA-MB-231 cells (Fig 8*A*), but intracellular levels of IGFBP-6 did not decrease (Fig. 8*B*), suggesting that intracellular IGFBP-6 is stabilized by cells. Extracellular levels of IGFBP-6 were measured by ELISA, and an 80% decrease in extracellular IGFBP-6 was observed (Fig. 8*B*). Interferon beta was not induced by treatment with siRNA in the 231 cells. However, levels of Cyclin B1, ARNT and HMMR substantially decreased (Fig. 8*C*). These results demonstrate that IGFBP-6 promotes expression of cyclin B1 in breast cancer cells. IGFBP-6 has been studied in the MDA-MB-231, where it was associated with increased proliferation and decreased migration (14). MDA-MB-231 cells did not recover after irradiation at their reported EC50 following knockdown of IGFBP-6, as interferon signaling was not activated (Fig. S11).

## Discussion

In this study, IGFBP-6 was identified as a regulator of cell cycle progression and interferon signaling using a proteomics approach after IGFBP-6 knockdown in Estrogen Receptor-positive T47D breast cancer cells. These results were validated for a subset of proteins identified for each of these cellular processes in T47D cells. Further analysis of these pathways in MDA-MB-231 cells, a model of triple-negative breast cancer, showed that knockdown of IGFBP-6 also resulted in a decrease in expression of the G2/M transition cyclin B1but no induction of ISGs was observed. It was also shown that knockdown of IGFBP-6 conferred resistance to irradiation in the T47D cells and not in the MDA-MB-231 cells. When comparing the relative levels of IGFBP-6 transcript and the intra- and extracellular accumulation across multiple cell lines, the level of transcript is not predictive for the level of intra- or extracellular IGFBP-6, suggesting the cellular modulation by IGFBP-6 is dependent on the accumulation and localization of IGFBP-6.

Previous reports demonstrate that IGFBP-6 confers improved outcomes for breast cancer patients (1, 2, 3). Consistent with these reports, our data show that knockdown of IGFBP-6 resulted in increased proliferation rates for T47D breast cancer cells. Proteomic and signaling pathway analysis identified increased expression of proteins and pathways associated with interferon signaling. Due to this increase, it was hypothesized that increased interferon-related signaling may mimic the Interferon-related DNA Damage Signature (IRDS) state where low chronic, levels of IFNb can protect cancer cells against DNA damaging therapeutics (15, 16, 17, 18, 19). The results from irradiation experiments revealed that reduced expression of IGFBP-6 improves recovery after irradiation exposure in T47D cells while MDA-MB-231 breast cancer cells that do not have increased interferon signaling markers do not have improved recovery. Furthermore, increases in the percentage of cells in G1 is associated with resistance to irradiation (44) and our results show that IGFBP-6 knockdown increased accumulation of cells in G1 compared to controls.

Disruption of the cell cycle has been reported to induce interferons through activation of the cGAS-STING pathway (23, 24, 46, 48). Differences in interferon signaling between the two cell lines used for IGFBP-6 knockdown studies revealed potential mechanisms of action for the regulation of interferon signaling by IGFBP-6. Data in this contribution suggest that the induction of interferon signaling is not associated with cGAS-STING activation resulting from cell cycle disruption because T47D breast cancer cells do not express cGAS or STING (48), and interferon signaling was not induced in cGAS-STING-positive MDA-MB-231 breast cancer cells. Other disruptions to the cell cycle can activate interferon signaling such as inhibition of Aurora A, resulting in activation of RIG-I (49). In this study, there is a decrease in proteins associated with events in the G2/M phase and an increase in ISGs, suggesting a link between the two. Further study is needed to determine the link between the cell cycle disruptions associated with IGFBP-6 knockdown and interferon signaling in breast cancer cells. It should be noted that this study was conducted *in vitro* and cannot account for recruitment or evasion of the immune system *in vivo*.

While the phosphoproteomics analysis did not reveal the activation of specific pathways that are known to be involved in both cell cycle regulation and interferon signaling, our previous work describing the cooperation of IGFBP-6 and progesterone receptor (PR) in breast cancer cell regulation may suggest that PR is the link that connects IGFBP-6 to interferon signaling. The PR-B isoform represses IFNb action in breast cancer cells (cite this study), and IGFBP-6 is a regulator of PR-B stability. Further study is needed to determine if PR-B specifically requires IGFBP-6 to repress IFN signaling. Progesterone is known to promote cell cycle entry and progression (50, 51, 52, 53) and increases IGFBP-6 levels intra and extracellularly. The results presented in this study suggest that IGFBP-6 ensures cell cycle progression and may mediate cell cycle progression in response to progesterone. Cells with low IGFBP-6 proliferate faster despite the changes in cell cycle distributions, suggesting that IGFBP-6 may have a role in the timing of cell cycle events. Knockdown of IGFBP-6 may increase the relative amount of time spent in G1 and decrease the amount spent in G2. Timing of cell cycle events, including expression of G2/M proteins, is regulated by FOXM1 and the DREAM complex (53). One possible mechanism to explain the observed cell cycle alterations is the regulation of the DREAM complex by IGFBP-6. Wee1 and CDK1 phosphorylation were decreased further, suggesting that IGFBP-6 may regulate exit out of G1 (Fig. 4*C*). An unexpected finding was that most T47D cells, regardless of siRNA treatment, were not mitotic at the time of measurement. (Fig. S6). This may suggest that there is either a large population of senescent or quiescent cells or that these cells spend a relatively long time in G0 (Fig. S5).

One observation from the current study that highlights the complexity of IGFBP-6 regulation is the lack of consistency between transcript level and the accumulation of intra- and extracellular IGFBP-6. It was apparent in our studies that the level of transcript measured in any particular cell line did not necessarily correlate with the accumulation of intra- or extracellular IGFBP-6 (Figs. S9 and S10). Additionally, a nearly 90% knockdown of IGFBP-6 transcript in the MDA-MB-231 cells did not result in a decrease in intracellular IGFBP-6, yet a significant reduction in extracellular IGFBP-6 was observed. Furthermore, IGFBP-6 knockdown was more efficient with regard to extracellular IGFBP-6 in MDA-MB-231 cells when compared to T47D cells. Taken together with the results of the MDA-MB-231 cells, this suggests that IGFBP-6 regulates the cell cycle in an extracellular manner. Additionally, these results suggest that breast cancer cells may stabilize intracellular IGFBP-6. Given these localization-dependent reductions of IGFBP-6, it is hypothesized that disruption of intracellular IGFBP-6 may be required to activate interferon signaling. Further study is needed to determine the localization-specific roles of IGFBP-6. An immunoprecipitation mass spectrometry experiment should be done to determine novel protein-protein interactions.

From this study, we can conclude that IGFBP-6 is a regulator of the cell cycle in breast cancer. Knockdown of IGFBP-6 results in decreases in Cyclin B1 and decreases in the percentage of cells in G2/M. In T47D cells, knockdown of IGFBP-6 also results in an induction of interferon signaling and resistance to radiation. Further study is required to understand the mechanisms by which IGFBP-6 participates in breast cancer cells.

## Experimental procedures

### Cell culture

Many of the methods have been previously described (54). T47D, MCF-7, MDA-MB-231, and MDA-MB-468 breast cancer cells were acquired from ATCC. T47D-Y cells were a generous gift from Dr Carol Lange (University of Minnesota). HEK293T cells were a generous gift from Dr John Nolan (Cellarcus Biosciences). Cells were maintained in DMEM supplemented with 10% fetal bovine serum, 1 mM sodium pyruvate, and 2 mM L-glutamine (Life Technologies). Cells below passage 15 were used except for T47D Y cells, which were at a passage between 45 and 50. All nucleotide and protein purifications were performed on cells at similar confluency.

### Total RNA extraction and quantitative real-time PCR analysis

Total RNA was extracted using the PureLink RNA Mini Kit (Life Technologies). Synthesis of cDNA was done with 1 μg of extracted RNA using the High-Capacity RNA-to-cDNA Kit (Life Technologies) and used as a template for quantitative real-time PCR (RT-qPCR) reactions. RT-qPCR was performed with SYBR Green Master Mix (Life Technologies) and the 7300 Real-Time PCR system (Bio-Rad, Hercules, CA). Human RPL30 was used as an internal control to normalize for mRNA in RT-qPCR reactions. The following primer pairs were used: IGFBP-6 (Forward 5′-CGAGGGGCTCAAACACTCTA-3′, Reverse 5′-CATCCGATCCACACACCAG-3′), IFNb (Forward 5′-GGCAGTATTCAAGCCTCCCA-3′, Reverse 5′-ACTGCAACCTTTCGAAGCCT-3′), and RPL30 (Forward 5′- ACAGCATGCGGAAAATACTAC-3′, Reverse 5′- AAAGGAAAATTTTGCAGGTTT-3′).

### Phosphopeptide enrichment and mass spectrometry

Total protein from each sample was reduced, alkylated, and purified by chloroform/methanol extraction prior to digestion with sequencing-grade trypsin and LysC (Promega). The resulting peptides were labeled using a tandem mass tag 11-plex isobaric label reagent set (Thermo Scientific), combined into a single multiplex group, then enriched using High-Select TiO2 and Fe-NTA phosphopeptide enrichment kits (Thermo Scientific, Rockford, IL) following the manufacturer’s instructions. Both enriched and un-enriched labeled peptides were separated into 46 fractions on a 100 × 1.0 mm Acquity BEH C18 column (Waters) using an UltiMate 3000 UHPLC system (Thermo Scientific) with a 50 min gradient from 99:1 to 60:40 buffer A:B ratio under basic pH conditions, then consolidated into 18 super-fractions for both the enriched and un-enriched sample sets. Buffer A was composed of a solution of10 mM ammonium hydroxide and 0.5% acetonitrile, and Buffer B was 10 mM ammonium hydroxide, 99.9% acetonitrile. Both buffers were adjusted to pH 10 for offline separation.

Each super-fraction was then further separated by reverse phase XSelect CSH C18 2.5 um resin (Waters) on an in-line 150 × 0.075 mm column using an UltiMate 3000 RSLCnano system (Thermo Scientific). Peptides were eluted using a 75 min gradient from 98:2 to 60:40 buffer A:B ratio. For this separation, buffer A was composed of 0.1% formic acid, 0.5% acetonitrile and B was composed of 0.1% formic acid, 99.9% acetonitrile. Eluted peptides were ionized by electrospray (2.4 kV) followed by mass spectrometric analysis on an Orbitrap Eclipse Tribrid mass spectrometer (Thermo Scientific, Rockford, IL) using multi-notch MS3 parameters. MS data were acquired using the FTMS analyzer in top-speed profile mode at a resolution of 120,000 over a range of 375 to 1500 m/z. Following CID activation with normalized collision energy of 31.0, MS/MS data were acquired using the ion trap analyzer in centroid mode and normal mass range. Using synchronous precursor selection, up to 10 MS/MS precursors were selected for HCD activation with normalized collision energy of 55.0, followed by acquisition of MS3 reporter ion data using the FTMS analyzer in profile mode at a resolution of 50,000 over a range of 100 to 500 m/z.

### Phosphoproteome data analysis

Proteins were identified and reporter ions quantified by searching the UniprotKB database restricted to *Homo sapiens* (March 2024) using MaxQuant (Max Planck Institute, version 2.1.4.0) with a parent ion tolerance of 3 ppm, a fragment ion tolerance of 0.5 Da, a reporter ion tolerance of 0.001 Da, trypsin/P enzyme with two missed cleavages, variable modifications including oxidation on M, Acetyl on Protein N-term, and phosphorylation on STY, and fixed modification of Carbamidomethyl on C. Protein identifications were accepted if they could be established with less than 1.0% false discovery. Proteins identified only by modified peptides were removed. Protein probabilities were assigned by the Protein Prophet algorithm (55). TMT MS3 reporter ion intensity values are analyzed for changes in total protein using the unenriched lysate sample. Phospho(STY) modifications were identified using the samples enriched for phosphorylated peptides. The enriched and un-enriched samples are multiplexed using two TMT11-plex batches, one for the enriched and one for the un-enriched samples. N = 4 for cells treated with the scrambled negative control, N = 3 for cells treated with siRNA one and N = 4 for cells treated with siRNA 3. siRNA two was not submitted for phosphoproteomics analysis.

Following data acquisition and database search, the MS3 reporter ion intensities were normalized using ProteiNorm (56). The data were normalized using RLR (Robust Linear Regression) and analyzed using proteoDA to perform statistical analysis using Linear Models for Microarray Data (limma) with empirical Bayes (eBayes) smoothing to the standard errors (57, 58, 59).

A similar approach is used for differential analysis of the phosphopeptides, with the addition of a few steps. The phosphosites were filtered to retain only peptides with a localization probability > 75%, filter peptides with zero values, and log2 transformed. Limma was also used for differential analysis. Proteins and phosphopeptides with an FDR-adjusted *p*-value < 0.05 and an absolute fold change > 1.5 were considered significant. Data were deposited in massIVE. ftp://massive-ftp.ucsd.edu/v10/MSV000098630/

### Kinase-substrate enrichment analysis (KSEA)

KSEA was done using the online tool KSEAapp (https://casecpb.shinyapps.io/ksea/), whose general method is described by Wiredja *et al.* (60). Phosproteome data was compared against the phosphosite plus database (61). *p* values were cutoff at 0.05, and substrate counts were cutoff at 5.

### Gene set enrichment analysis

The general method for gene set enrichment analysis is described by Subramanian *et al.* (62). Proteins were ranked based on their relative fold change compared to the scrambled negative control. GSEA was done using the fgsea package in R (63). The Reactome pathway data set was used to match proteins to a pathway in the analysis (64).

### Protein interaction network visualization

All protein interaction networks were made in Cytoscape Version 3.10.3 (65). Protein networks were generated from proteome and phosphoproteome data using StringApp 2.0 (66), which looks up protein-protein interactions from STRING (67). Visualization of phosphosites or fold changes was done with the Omics Visualizer App (68). Clustering was done using Markov (MCL) clustering built into StringApp.

### Immunoblot analysis

Cells were lysed with RIPA buffer containing protease and phosphatase inhibitor cocktails (Life Technologies). After lysis cells were centrifuged at 9000*g* for 20 min at 4 °C and the supernatant was collected. Protein concentrations were determined by BCA assay (Thermo Scientific, Rockford, IL). 10 to 20 μg of protein was resolved using Bolt 4 to 12% Bis-Tris Plus gels and dry transferred to a PVDF membrane with the iBlot2 system (Life Technologies). Membranes were blocked in 1X Tris-buffered saline 0.1% Tween 20 (TBST) and either 10% Bovine Serum Albumin (BSA) (Thermo Scientific) for phospho-proteins or 5% fat-free milk for all other protein targets. Membranes were then washed in 1X TBST three times, primary antibody was added and allowed to incubate overnight at 4 °C. Proteins were incubated at a ratio of 1:1000 in 5% BSA in TBST for phosphoproteins or in 5% milk in TBST for other proteins. Antibodies used are as follows: IGFBP-6 (#ab219560, Abcam, Waltham, MA), MX1 (#37849 Cell Signaling), MDA5 (#5321 Cell Signaling), IRF9 (#76684 Cell Signaling), ISG15 (#2758 Cell Signaling), Phospho-STAT1 (Y701) (#7649 Cell Signaling), STAT1 (#14994 Cell Signaling), Phospho-STAT2 (Y690) (#88410 Cell Signaling), STAT2 (#72604 Cell Signaling), Cyclin B1 (#12231 Cell Signaling), Aurora A (#14475 Cell Signaling), CDC20 (#14866 Cell Signaling), HMMR (#55463 Cell Signaling), ARNT (HIF1-β) (#5537 Cell Signaling), and Beta Actin (#sc 47778, Santa Cruz Biotechnology). After incubation with primary antibodies, membranes were washed three times with 1X TBST then incubated with either anti-rabbit IgG conjugated to horseradish peroxidase (#7074, Cell Signaling Technology) or anti-mouse IgG conjugated to horseradish peroxidase (#7076, Cell Signaling Technology) for 1 h at room temperature with a dilution ratio of 1:5000. Membranes were then washed three times with 1X TBST before Supersignal chemiluminescence reagent (Thermo Scientific) was added and detected using Gel Doc XR ChemiDoc imaging system (BioRad) followed by quantification using ImageLab software (BioRad). Restore plus Western blot buffer (Thermo Scientific) was used to strip membranes of antibodies prior to probing for other targets.

### siRNA knockdown

Knockdown of IGFBP-6 was done in DMEM supplemented with 10% fetal bovine serum, 1 mM sodium pyruvate, and 2 mM L-glutamine (Life Technologies). Reverse transfections were done with lipofectamine RNAiMAX (Life Technologies). The following sequences were used to perform knockdowns: siRNA 1 (Sense 5′-GGAGAAUCCUAAGGAGAGUtt-3′, Antisense 5′-ACUCUCCUUAGGAUUCUCCtc-3′), siRNA 2 (Sense 5′-GAGGAGAAUCCUAAGGAGAtt, Antisense 5′-UCUCCUUAGGAUUCUCCUCtg), and siRNA 3 (Sense 5′-GAAUCCUAAGGAGAGUAAAtt-3′, Antisense 5′-UUUACUCUCCUUAGGAUUtc-3′). Silencer Negative control #1 (Life Technologies) was used as a negative control. For all knockdowns, a 120 nM dose of siRNA was used. Cells were then incubated for 48 h before performing the corresponding assay. For Western blot, 250,000 cells per well were plated in a 6-well plate for 48-h. Cells were then detached with 0.05 trypsin. Suspended in maintenance media and centrifuged at 1000 rpm for 5 min. The resulting pellet was then washed with 1X PBS and recentrifuged. After aspirating the PBS, the resultant cell pellet was flash frozen at −80 C. Cell pellets were then stored at −80C until use. For proliferation, FUCCI reporter, and senescence assays, 12,500 T47D cells and 5000 MDA-MB-231 cells were used in a 96 well plate. The concentrations of siRNA and transfection reagent remained the same.

### Extracellular IGFBP-6 measurement

Media was collected from each of the knockdowns and stored at −80 °C. Cells were collected and 15 uL of suspended cells were collected. Cells were counted on a hemocytometer after staining with trypan blue. Media was concentrated using Vivaspin centrifugal concentrators (Sartorius, Göttingen, Germany). On average, Measurement of secreted IGFBP-6 was done with an IGFBP-6 ELISA kit (Thermo Scientific, Rockford, IL) as described by the manufacturer. IGFBP-6 concentrations were normalized by total protein after measurement by the BCA Assay.

For Western blot, media were collected, stored, and concentrated as described above. Protein content was measured using a BCA Assay. Cells were then analyzed by Western blot as described above.

### Proliferation assay

96 well plates were used to measure proliferation in an Incucyte Live Cell Imager (Sartorius). Confluency was measured using phase contrast and images were analyzed using the basic analyzer. Proliferation was measured for 5 days for the T47D cells and 3 days for the 231 cells.

### Cell cycle analysis

T47D cells were transduced with Incucyte Cell Cycle Green/Red Lentivirus Reagent for 24 h and transduced cells were selected with media containing 2 mg/ml puromycin for 5 days. These cells will be referred to as T47D Fucci cells. After treatment with siRNA for 48 h, cells were imaged using an Incucyte Live Cell Imager (Sartorius).

### Radiation resistance measurement

After knockdown of IGFBP-6, cells were irradiated with X-rays using a MultiRad350 (Faxitron). T47D cells were irradiated at a dose of 4Gy, and 231 cells at a dose of 9Gy according to their published EC50 doses (45). Following irradiation, the media was switched to media containing siRNA and transfection reagent at the same concentration as with the knockdown. Proliferation was then measured and analyzed on the Incucyte as described above.

### Statistical analysis

All statistical analysis was performed using R. Statistical analysis included ANOVA with Tukey’s *post hoc* test. Repeated measures ANOVA were used for proliferation data. Data were tested for a normal distribution using the Shapiro-Wilk test. Kruskal Wallis Tests were done if data did not meet the assumptions for ANOVA with Dunn’s test for *post hoc* analysis. Differences were considered significant if *p* ≤ 0.05. All error bars are the standard error of the mean. Outliers were removed if values were identified as outliers on a boxplot in R. All values of N in the caption are for biological replicates.

## Data availability

Phosphoproteomics data was uploaded to massIVE and can be found at ftp://massive-ftp.ucsd.edu/v10/MSV000098630/. All other data is available upon request to the corresponding author.

## Supporting information

This article contains supporting information.

## Conflict of interest

The authors declare the following financial interests/personal relationships which may be considered as potential competing interests: Kevin Houston reports equipment, drugs, or supplies and writing assistance were provided by University of Arkansas for Medical Sciences. Kevin Houston reports equipment, drugs, or supplies was provided by University of Minnesota Twin Cities. If there are other authors, they declare that they have no known competing financial interests or personal relationships that could have appeared to influence the work reported in this paper.

## Supporting information

IGFBP6 Paper 2 Supplementalrev 2025_12_15
