# Supplementary material for: IGFBP-6 regulates breast cancer cell cycle progression by promoting exit out of G1
Source: J Biol Chem. 2025 Dec 17;302(2):111069. doi: 10.1016/j.jbc.2025.111069 (PMC12860944; doi:10.1016/j.jbc.2025.111069)
Supplement: IGFBP6 Paper 2 Supplementalrev 2025_12_15 [file mmc1.pptx]

## Slide 1
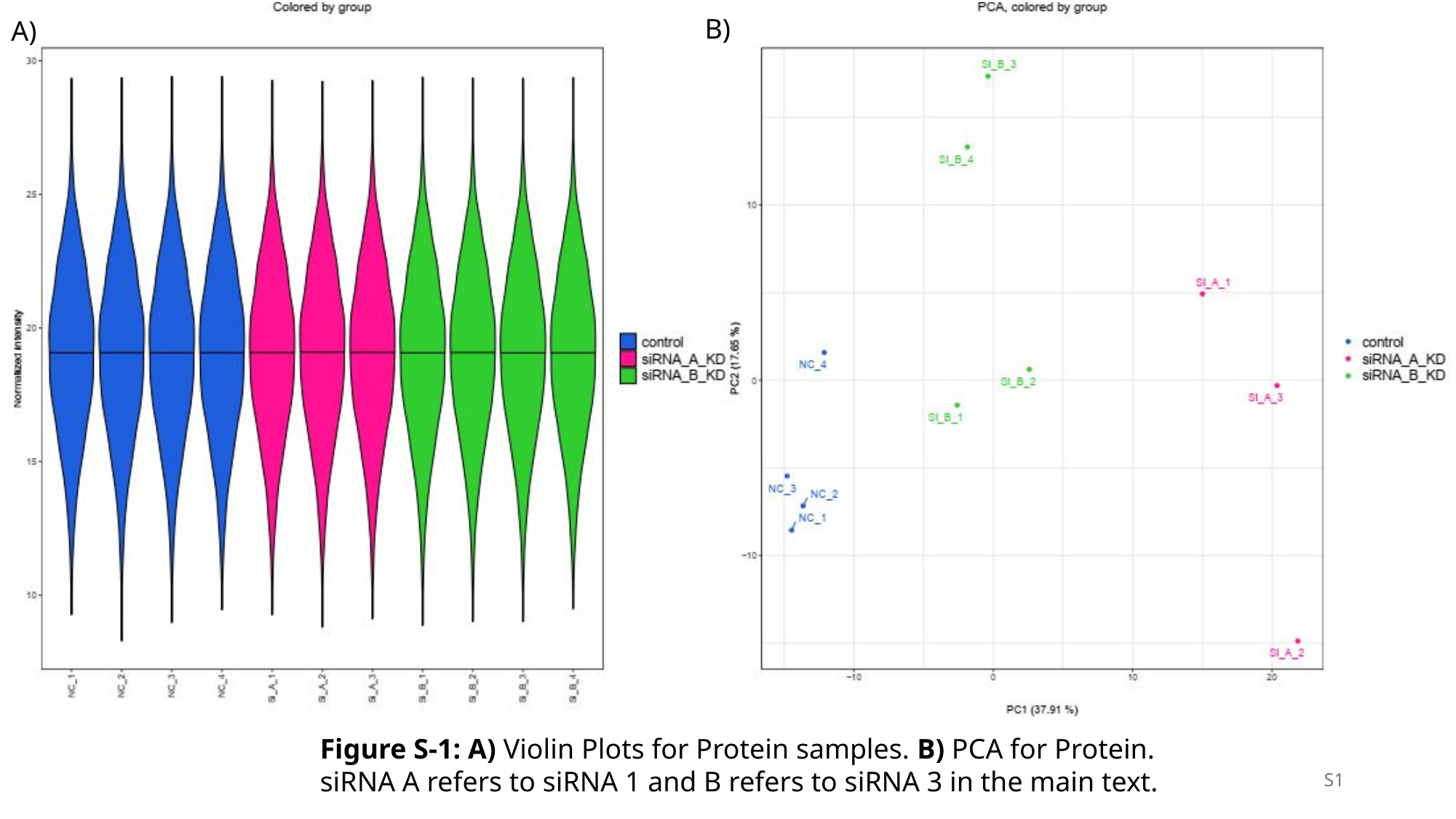

B)
A)
Figure S-1: A) Violin Plots for Protein samples. B) PCA for Protein.
siRNA A refers to siRNA 1 and B refers to siRNA 3 in the main text.
S1

## Slide 2
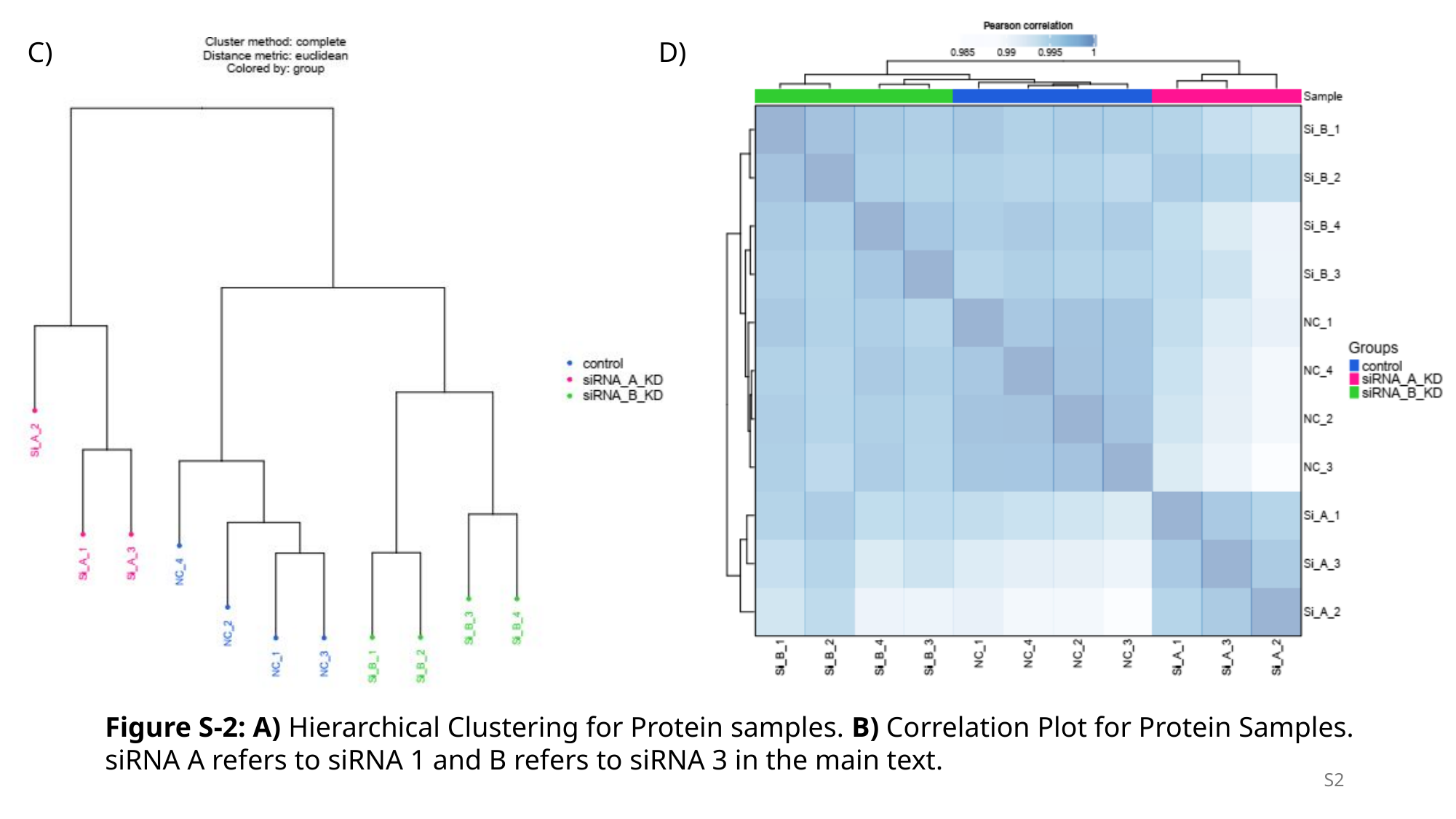

C)
D)
Figure S-2: A) Hierarchical Clustering for Protein samples. B) Correlation Plot for Protein Samples.
siRNA A refers to siRNA 1 and B refers to siRNA 3 in the main text.
S2

## Slide 3
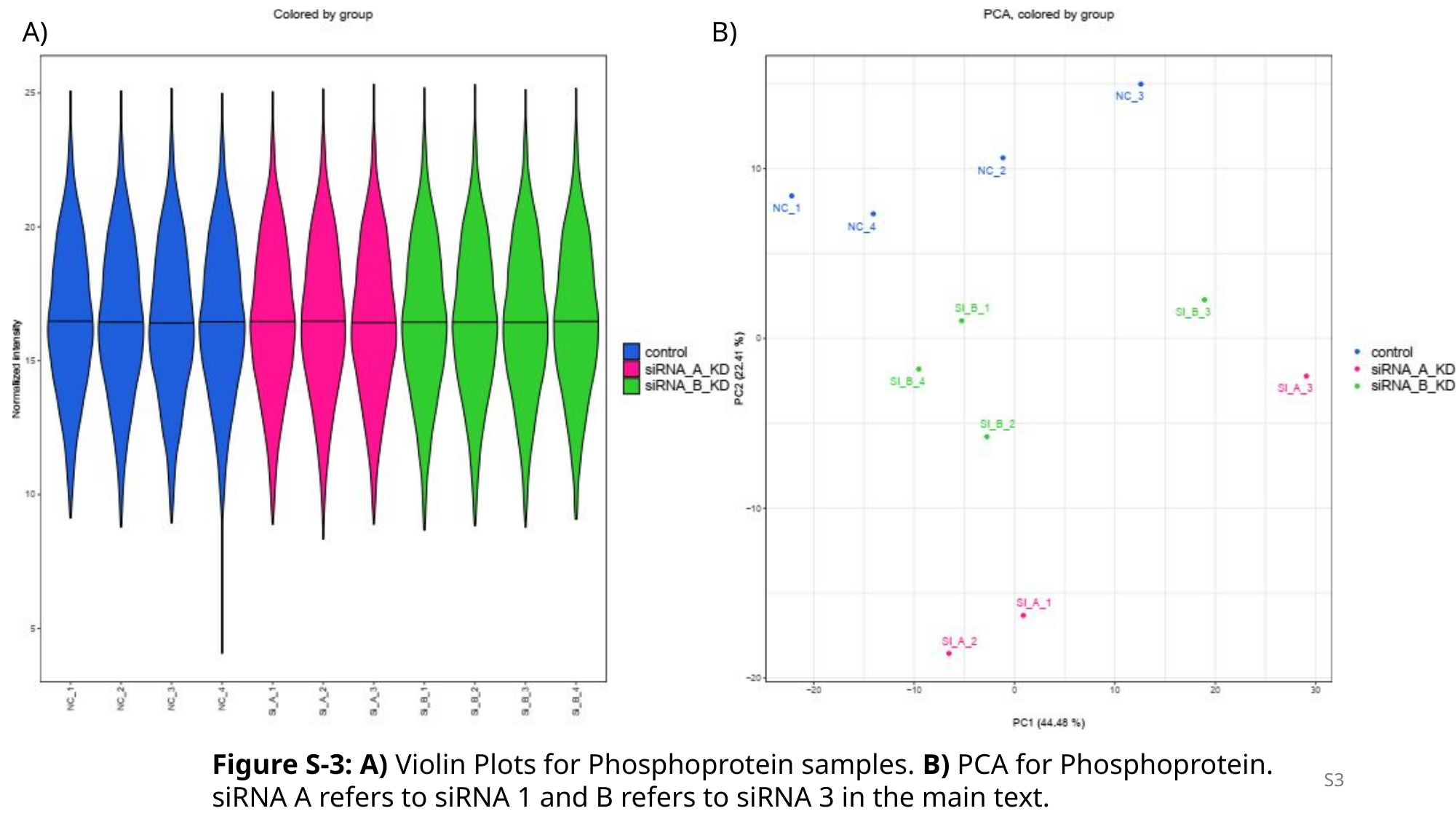

A)
B)
Figure S-3: A) Violin Plots for Phosphoprotein samples. B) PCA for Phosphoprotein.
siRNA A refers to siRNA 1 and B refers to siRNA 3 in the main text.
S3

## Slide 4
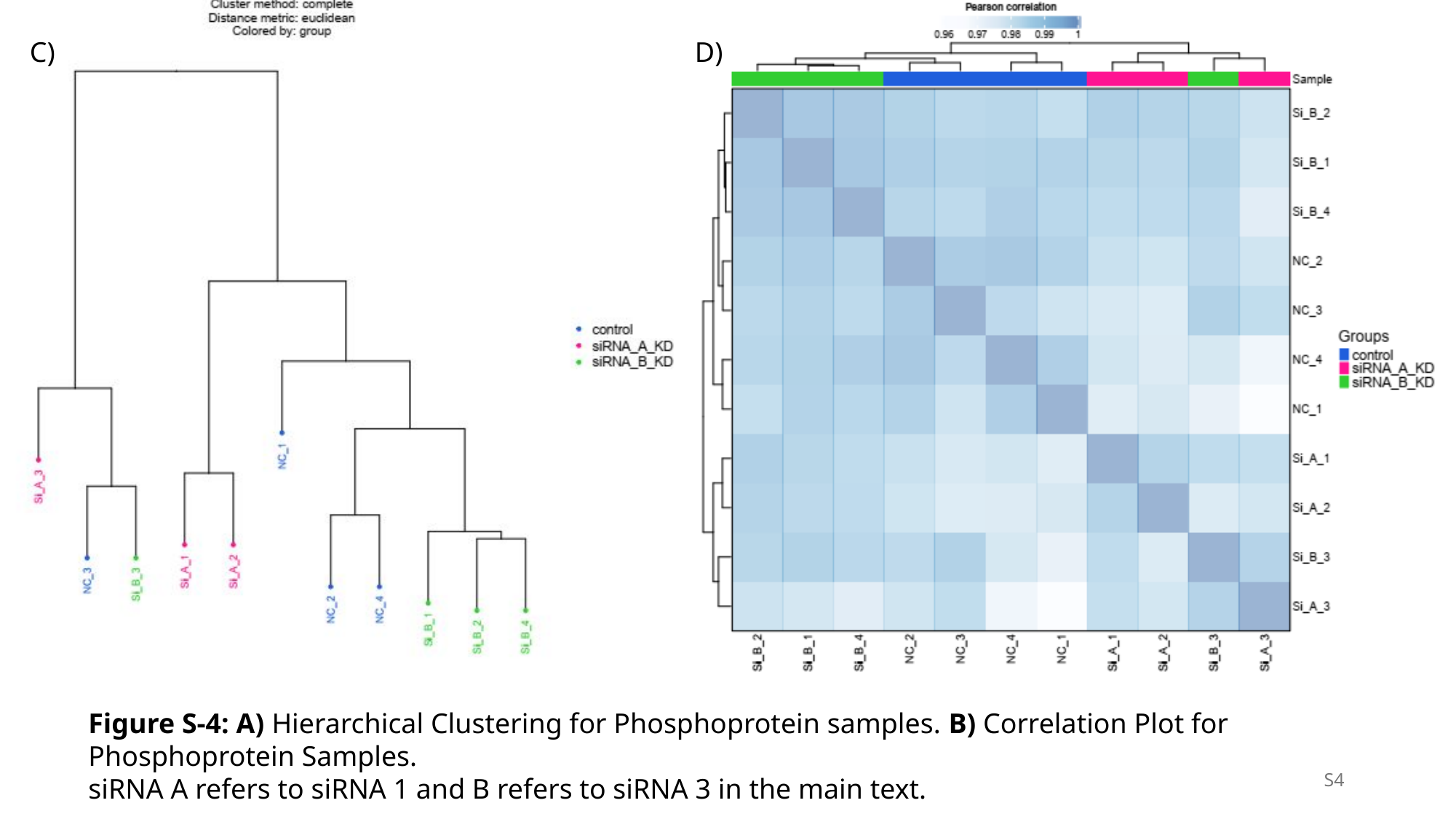

C)
D)
Figure S-4: A) Hierarchical Clustering for Phosphoprotein samples. B) Correlation Plot for Phosphoprotein Samples.
siRNA A refers to siRNA 1 and B refers to siRNA 3 in the main text.
S4

## Slide 5
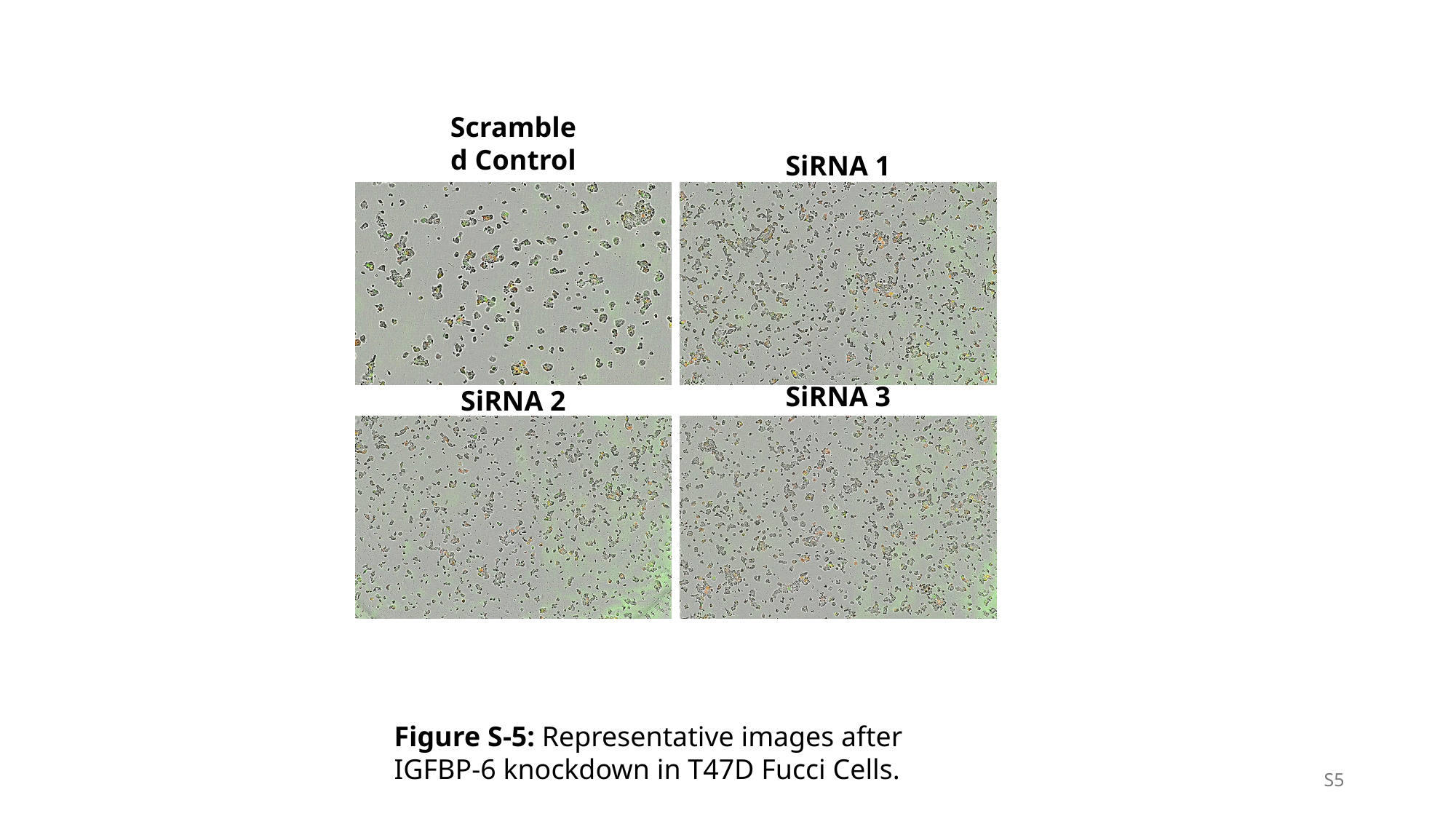

Scrambled Control
SiRNA 1
SiRNA 3
SiRNA 2
Figure S-5: Representative images after IGFBP-6 knockdown in T47D Fucci Cells.
S5

## Slide 6
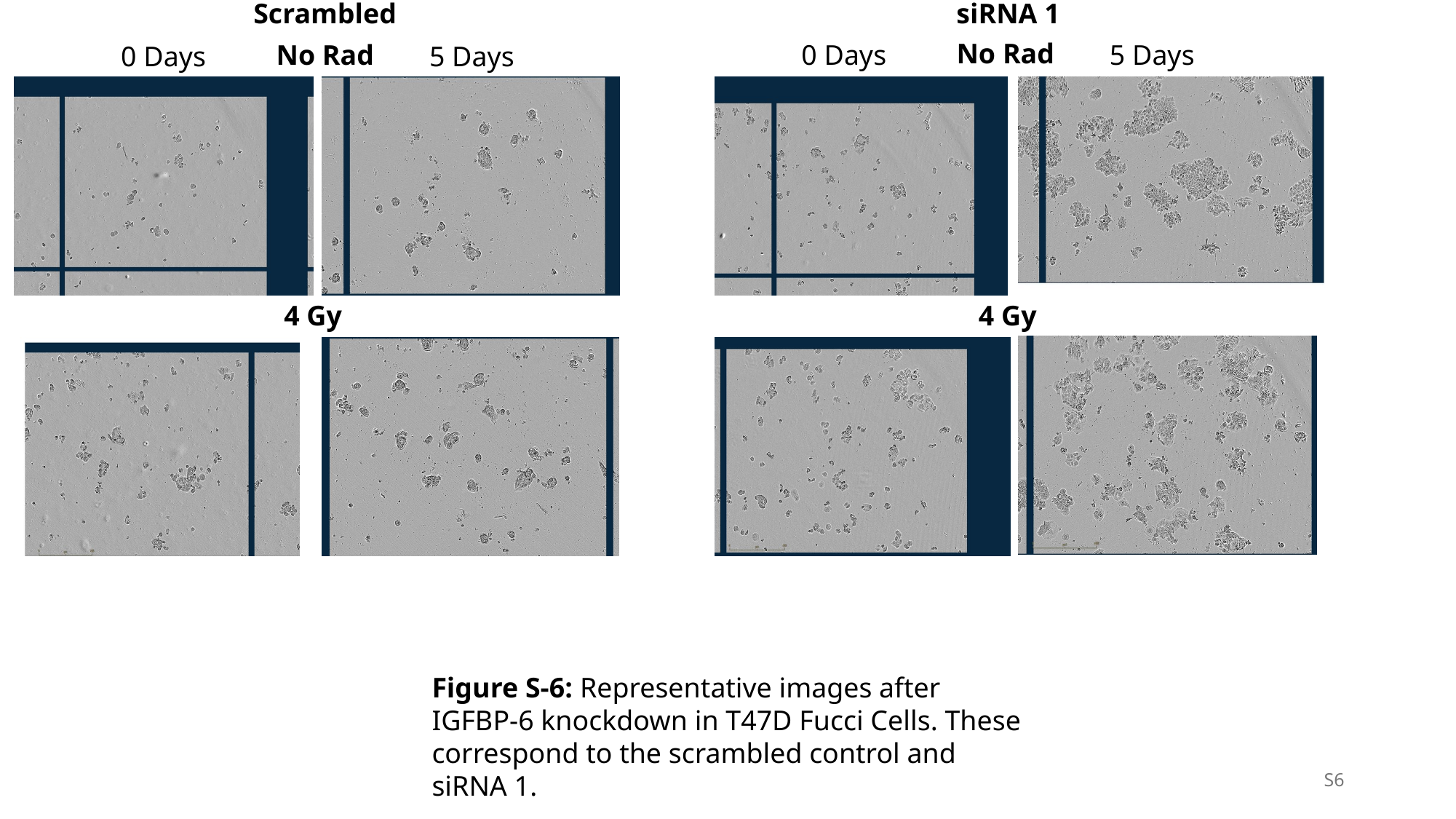

Scrambled
siRNA 1
No Rad
No Rad
0 Days
5 Days
0 Days
5 Days
4 Gy
4 Gy
Figure S-6: Representative images after IGFBP-6 knockdown in T47D Fucci Cells. These correspond to the scrambled control and siRNA 1.
S6

## Slide 7
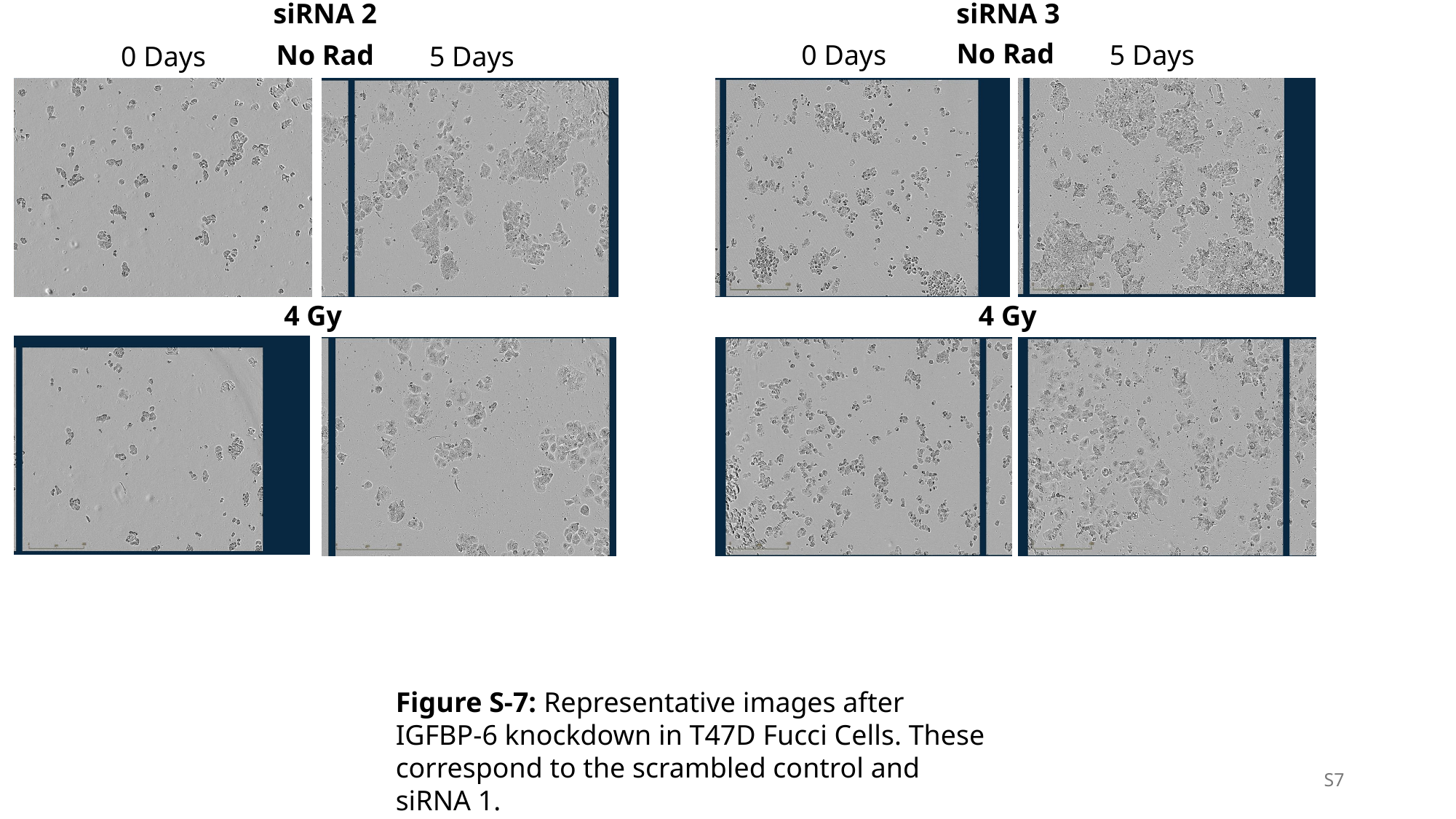

siRNA 2
siRNA 3
No Rad
No Rad
0 Days
5 Days
0 Days
5 Days
4 Gy
4 Gy
Figure S-7: Representative images after IGFBP-6 knockdown in T47D Fucci Cells. These correspond to the scrambled control and siRNA 1.
S7

## Slide 8
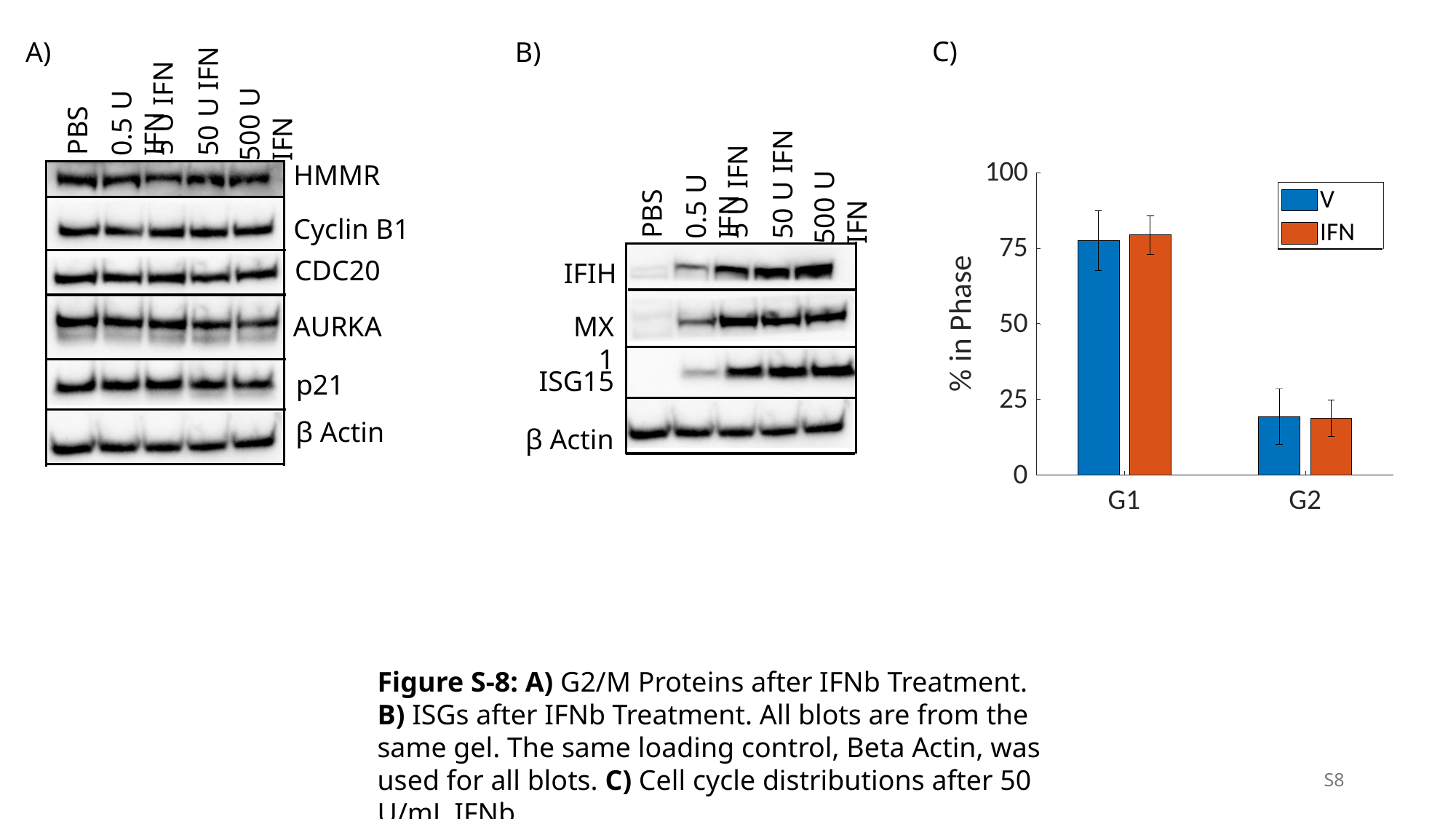

C)
A)
B)
5 U IFN
0.5 U IFN
50 U IFN
500 U IFN
PBS
HMMR
5 U IFN
0.5 U IFN
50 U IFN
500 U IFN
PBS
Cyclin B1
CDC20
IFIH
AURKA
MX1
ISG15
p21
β Actin
β Actin
Figure S-8: A) G2/M Proteins after IFNb Treatment. B) ISGs after IFNb Treatment. All blots are from the same gel. The same loading control, Beta Actin, was used for all blots. C) Cell cycle distributions after 50 U/mL IFNb.
S8

## Slide 9
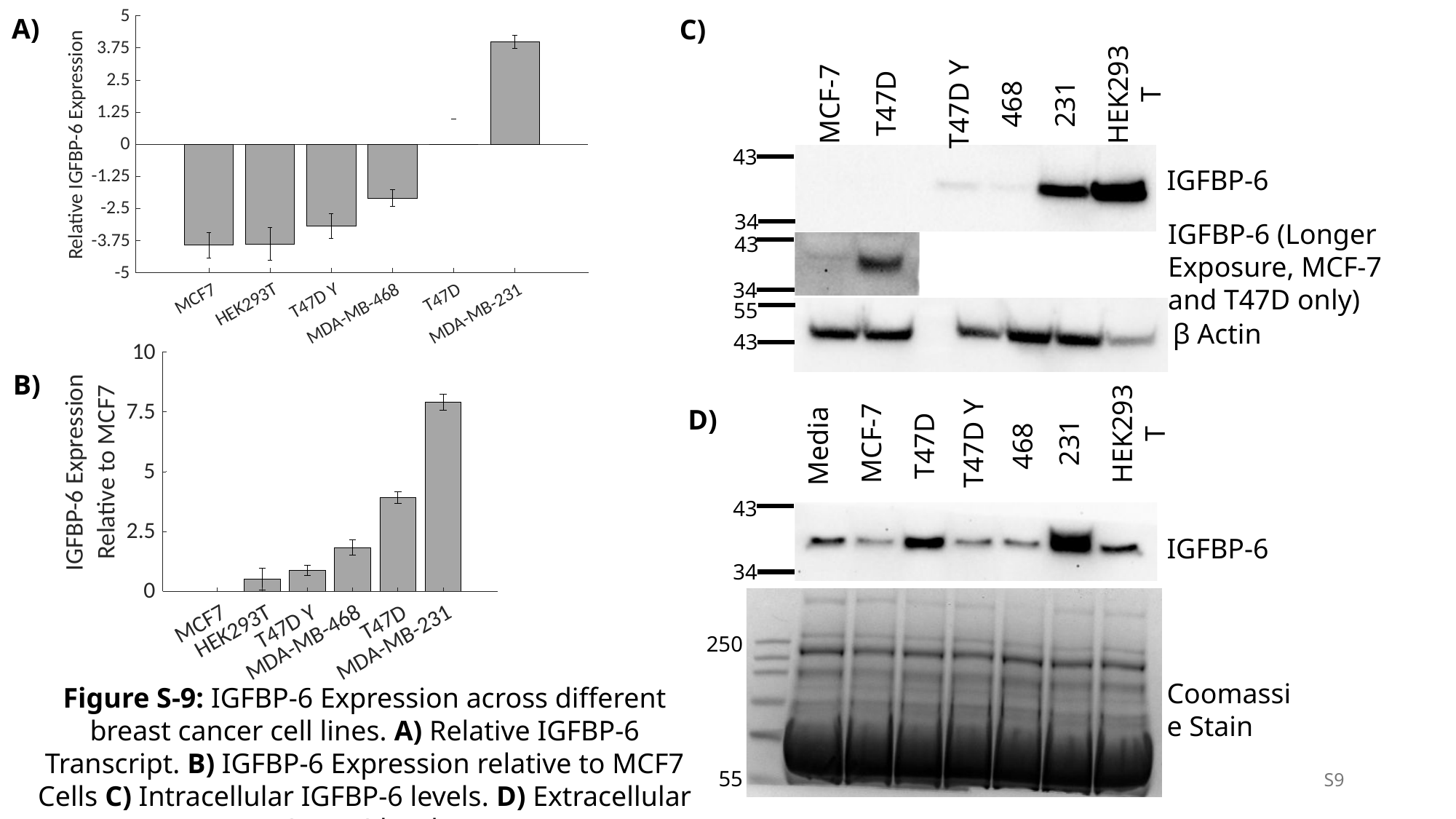

A)
C)
HEK293T
T47D
468
231
T47D Y
MCF-7
43
IGFBP-6
34
IGFBP-6 (Longer Exposure, MCF-7 and T47D only)
43
34
55
β Actin
43
B)
D)
HEK293T
231
T47D Y
MCF-7
Media
T47D
468
43
IGFBP-6
34
250
Coomassie Stain
Figure S-9: IGFBP-6 Expression across different breast cancer cell lines. A) Relative IGFBP-6 Transcript. B) IGFBP-6 Expression relative to MCF7 Cells C) Intracellular IGFBP-6 levels. D) Extracellular IGFBP-6 levels.
S9
55

## Slide 10
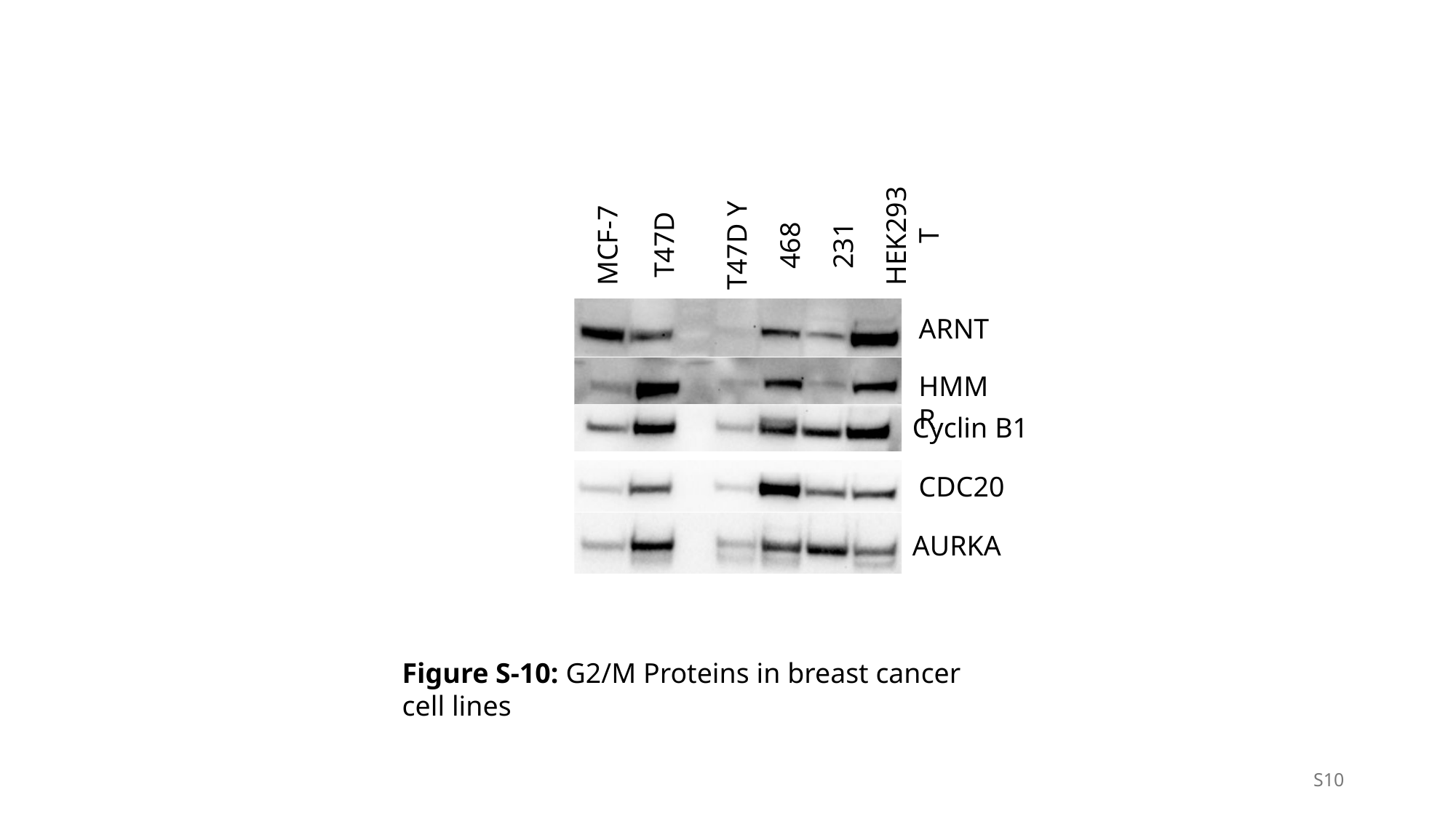

HEK293T
T47D
468
231
T47D Y
MCF-7
ARNT
HMMR
Cyclin B1
CDC20
AURKA
Figure S-10: G2/M Proteins in breast cancer cell lines
S10

## Slide 11
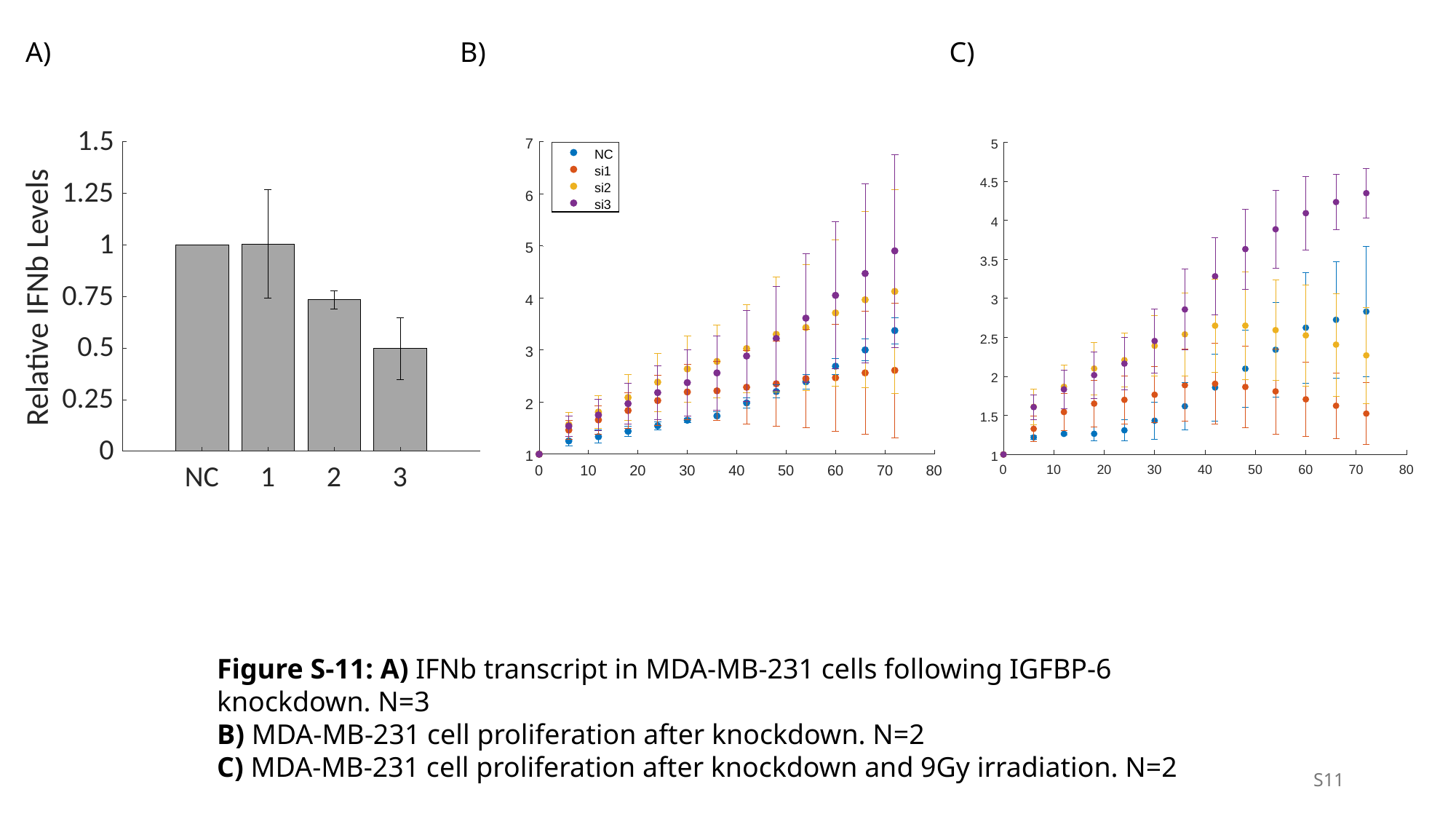

A)
B)
C)
Figure S-11: A) IFNb transcript in MDA-MB-231 cells following IGFBP-6 knockdown. N=3
B) MDA-MB-231 cell proliferation after knockdown. N=2
C) MDA-MB-231 cell proliferation after knockdown and 9Gy irradiation. N=2
S11
